# Complete mitochondrial genome of the glowbelly *Acropoma japonicum* (Perciformes: Acropomatidae) in the East China Sea

**DOI:** 10.1080/23802359.2019.1670109

**Published:** 2019-10-15

**Authors:** Yanming Sui, Yanqing Wu, Liguo Yang, Wenquan Sheng, Bianbian Zhang

**Affiliations:** Key Laboratory of East China Sea Fishery Resources Exploitation, Ministry of Agriculture, East China Sea Fisheries Research Institute, Chinese Academy of Fishery Sciences, Shanghai, China

**Keywords:** *Acropoma japonicum*, mitochondrial genome, phylogenetic analysis

## Abstract

The complete mitochondrial genome sequence of *Acropoma japonicum* is first described in this article. The total length of mitogenome is16,973 bp. It contains 13 protein-coding genes, 23 tRNA genes, and 2 ribosomal RNA genes. The overall base composition of H-strand is 27.64% A, 29.49% C, 26.84% T, and 16.03% G, with an A＋T bias of 54.48%. The phylogenetic analysis result showed that the *A. japonicum* and *Lutjanus peru* had a close relationship.

The glowbelly *Acropoma japonicum* belongs to genus Acropoma in family Acropomatidae of order Perciformes. It is a kind of small fish which is endemic in the continental shelf from the West Pacific to the Indian Ocean. It is also an economically important fishery resource in Japan (Hatooka et al. 2002; Okuda et al. 2005; Shen et al. 2009).

The complete mitochondrial genome of *A. japonicum* first determined in this paper was expected to provide help on population genetics of *A. japonicum* and further molecular phylogenetic studies.

The sample of *A. japonicum* in this article was collected from the East China Sea (121°56′E and 30°52′N). The specimen was stored in the Key Laboratory of East China Sea Fishery Resources Exploitation, Ministry of Agriculture. According to genes from *A. japonicum*, such as partial sequence of 12S ribosomal (Accession: LC021232), 16S ribosomal RNA gene (Accession: DQ790843), cytochrome c oxidase subunit I (COI) gene (Accession: DQ648437), and cytochrome b (Cyt b) gene (Accession: AB104911) primers was designed and PCR amplification and sequencing were conducted.

The whole length of *A. japonicum* mitogenome was 16,973 bp and submitted in GenBank (Accession No. MH924166). The nucleotide composition of the heavy strand was 27.64% for A, 29.49% for C, 26.84% for T, and 16.03% for G, with a high A＋T bias of 54.48%. It contains 13 protein-coding genes, 23 tRNAs, and 2 rRNAs. Most genes were located on the heavy strand, but *ND6* and 8 tRNA genes (*tRNA^Gln^*, *RNA^Ala^*, *tRNA^Asn^*, *tRNA^Cys^*,*tRNA^Tyr^*, *tRNA^Ser^*, *tRNA^Glu^*, *tRNA^Pro^*) were encoded on the light strand. Most protein-coding genes initiated with ATG except for COI starting with GTG. It is also important to note that the majority of protein-coding genes (seven of 13 genes) is inferred to terminate with an incomplete stop codon T or TA– (ND2, COII, ATPase 6, COIII, ND3, ND4, and Cyt b), four protein-coding genes share the typical termination codon TAA (ND1, ATPase 8, ND4L, and ND5), COXI and ND6 use AGA,TAG as a stop codon, respectively. The length of 12S (located between *tRNA^Glu^* and *tRNA^Met^*) and 16S (located between *tRNA^Phe^* and *tRNA^Pro^*) rRNA genes were 955 bp and 1693 bp, respectively.

To investigate the phylogenetic relationship, we downloaded the mitochondrial genome sequences of 13 currently available Percomorphaceae for subsequent phylogenetic analysis with *Protopterus annectens* (JX568887) as an outgroup. The concatenated sequences of 13 protein-coding genes, 2 rRNAs genes, and 22 tRNAs genes were aligned with the ClustalW program (Larkin et al. 2007). Using the maximum likelihood (ML) method (Stamatakis 2006), the phylogenetic tree was constructed (Figure 1) by MEGA 6 (Tamura et al. 2013). The best-fitting model (GTR + I+G) was obtained as the optimization model by jModelTest (Posada 2008). The result indicating that the *A. japonicum* and *Lutjanus peru* had a close relationship (Figure 1).

**Figure 1.**
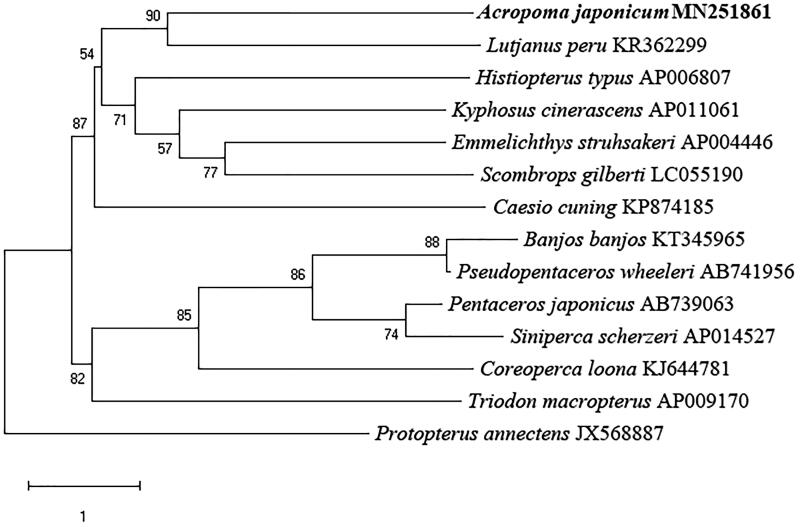
The phylogenetic tree based on the 13 protein-coding genes, 2 rRNAs genes, and 22 tRNAs genes of *A. japonicum*, *Scombrops gilberti, Howella brodiei*, *Banjos banjos*, *Pseudopentaceros wheeleri*, *Pentaceros japonicas*, *Histiopterus typus*, *Siniperca scherzeri*, *Coreoperca loona, Kyphosus cinerascens*, *L. peru*, *Caesio cuning*, *Emmelichthys struhsakeri*, *Triodon macropterus*, and the outgroup *P. annectens*. The bootstrap supports for maximum likelihood (ML) method was indicated at each branch.
